# The complete chloroplast genome sequence and phylogenetic analysis of an invasive plant *Solanum carolinense* Linnaeus (Solanaceae) in Korea

**DOI:** 10.1080/23802359.2026.2635843

**Published:** 2026-02-25

**Authors:** Hye Been Kim, Ju Eun Jang, Dong Chan Son, Eun Su Kang

**Affiliations:** Division of Forest Biodiversity Research, Korea National Arboretum, Pocheon, Republic of Korea

**Keywords:** Plastome, coding sequences, *Solanum*, phylogenetic relationship

## Abstract

*Solanum carolinense* Linnaeus, belonging to the family Solanaceae, is a perennial herb or subshrub. *S. carolinense* has become naturalized in Korea as an invasive species, forming a stable population that has grown naturally with native plants for more than 10 years. However, its chloroplast genome structure and complete sequence have not yet been reported. Therefore, we determined the complete chloroplast genome sequence of *S. carolinense* using genome sequencing, assembly, and annotation. The total length of the chloroplast genome was 155,315 bp with a GC content of 37.6%. It featured a quadripartite structure (a large single-copy region, 86,160 bp; a small single-copy region, 18,459 bp; and two inverted repeat regions, 25,348 bp each). It contains 129 genes, including 84 coding sequences (CDSs), 37 tRNA genes, 8 rRNA genes, and one pseudogene. Phylogenetic analysis of 78 CDSs revealed that *S. carolinense* is closely related to *S. aridum* Morong and *S. hieronymi* Kuntze. These results provide a molecular foundation for phylogenetic and evolutionary studies of the genus *Solanum* and present a fundamental chloroplast genomic resource for future invasion biology research.

## Introduction

*Solanum carolinense* Linnaeus (1753), of subgenus *Leptostemonum* (Solanaceae), is a perennial herb or subshrub native to North America, growing in temperate biomes (POWO 2025). This species was introduced to the Republic of Korea in 1978 and, after naturalization, has spread widely, disturbed ecosystems, and is now recognized as an invasive alien plant managed at the national level (Kang et al. 2025).

Next-generation sequencing (NGS) technologies have facilitated the sequencing and analysis of chloroplast genomes (Nock et al. 2011; Li et al. 2015; Daniell et al. 2016). Chloroplast genome data are used in plant taxonomy, phylogeny, and evolution studies and provide valuable insights into the evolutionary history of taxa with complex lineages (Yang et al. 2013; Luo et al. 2021; Kim et al. 2023). In addition, chloroplast genome data are increasingly utilized to study invasive species. For example, the analyses of genetic diversity using chloroplast-derived simple sequence repeat (SSR) or single-nucleotide polymorphism (SNP) markers have played important roles in tracing the origins of invasive species, elucidating their dispersal pathways, and identifying genetic factors that facilitate their spread (Dowell et al. 2016; Hinsinger and Strijk 2017; Meyer et al. 2017; Winkler et al. 2019). Despite its ecological significance and invasiveness, the complete chloroplast genome of *S. carolinense* has not been reported, although partial sequence-based phylogenetic analyses exist (Bohs 2005; Levin et al. 2006; Weese and Bohs 2007; Stern et al. 2011; Zhang et al. 2013; Wahlert et al. 2015).

This study aims to sequence the complete chloroplast genome of *S. carolinense* and analyze its phylogenetic relationships with related taxa. The results of this study will provide a foundational resource for future research in phylogeny and invasive plant management.

## Materials and methods

**Figure 1.**
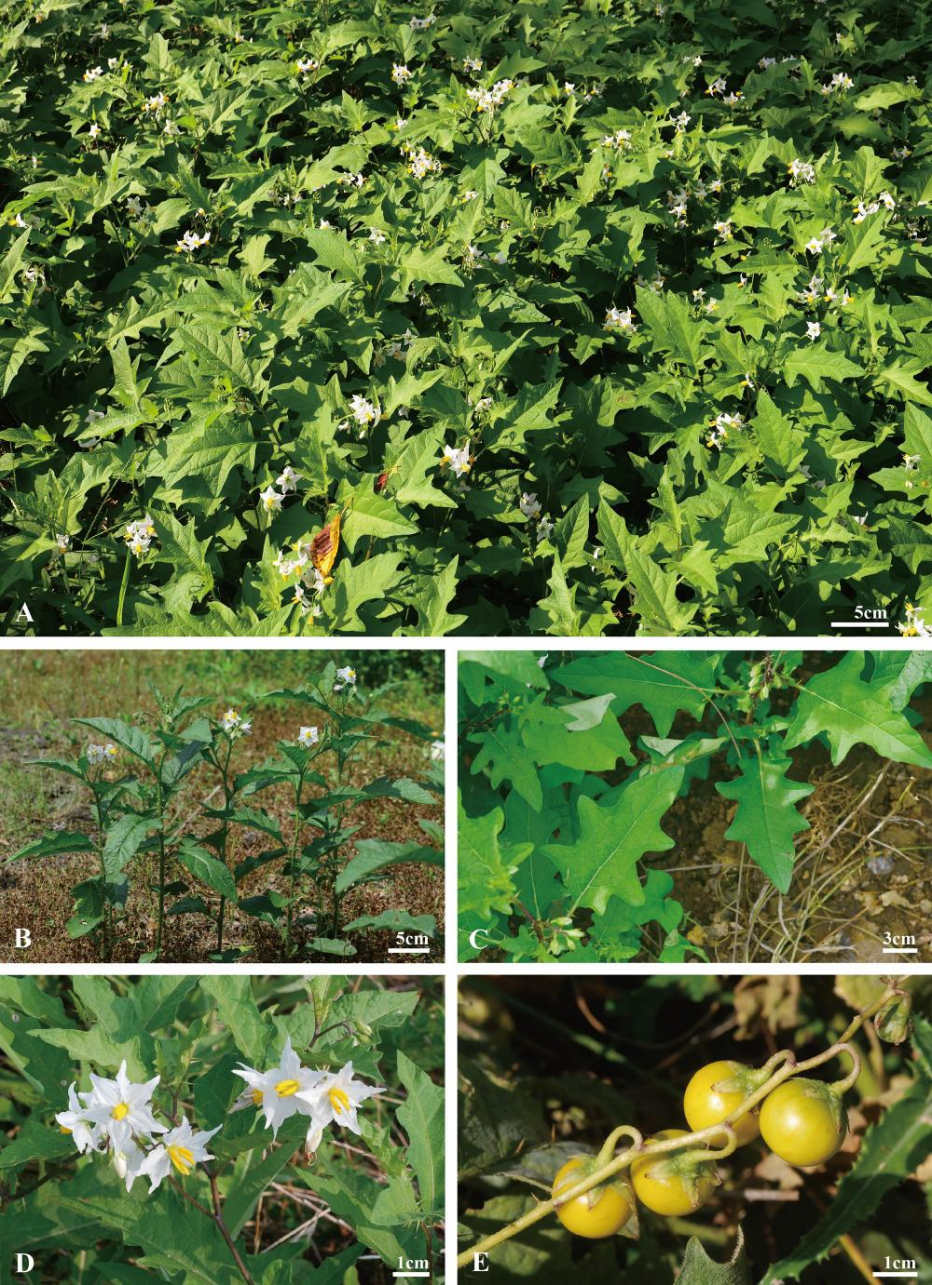
Photographs of *solanum carolinense* taken by jin sook kim, Seong Gwon Lee, and sa bum jang in yeonggwang-gun, jeollanam-do (A), jeju-si, jejudo island (B-E). Permission to use the photographs was obtained from jin sook kim, Seong Gwon Lee, and sa bum jang. (A) *S. carolinense* is a common weed found in vacant lots, grasslands, and Forest edges in South Korea. (B) Stem of *S. carolinense*, erect, sparsely pubescent, and armed with prickles. (C) Leaf of *S. carolinense*, ovate to elliptic or oblong, with sinuate margins and large prickles along the veins. (D) Flower of *S. carolinense*, radially symmetric, white to pale blue. (E) Fruit of *S. carolinense*, globose, and turning bright yellow when mature.

*S. carolinense* was identified and collected in Woljeong-ri, Gujwa-eup, Jeju-si, Jeju-do, the Republic of Korea (33°33’36.7" N, 126°47’25.7" E) (Figure 1) in August 2022 by Seong Gwon Lee. A voucher specimen was deposited at the herbarium of the Korea National Arboretum (KH) under voucher number KHB1662055 (http://www.nature.go.kr, Contact: Dong Chan Son, sdclym@korea.kr) (Figure S1). Fresh leaves were dried and preserved in silica gel for DNA extraction.

Total DNA was extracted using the DNeasy Plant Mini Kit (Qiagen, Hilden, Germany), following the manufacturer’s protocol. The extracted DNA was detected on a 2% agarose gel, and high-purity genomic DNA was sequenced using an Illumina MiSeq platform with a 2 × 301 bp paired-end reads, generating 6,936,450 reads. *De novo* plastome assembly was performed using NOVOPlasty v.4.3.5 with default parameters, except that the expected genome size was set to 150,000–200,000 bp. The final assembly of the complete cp genome was conducted using Geneious Prime v.2025.2.2 (Kearse et al. 2012). To verify the genome assembly, read coverage depth was assessed using the Draw_SequencingDepth.py script, as described by Ni et al. (2023). Finally, GeSeq (Tillich et al. 2017) was used to annotate the genome sequence. Gene arrangement was confirmed by comparing the chloroplast genome data of related species in the NCBI database (https://www.ncbi.nlm.nih.gov). The chloroplast genome map, including an intron structure map of cis- and trans-splicing genes, was visualized using CPGview (Liu et al. 2023).

For phylogenetic analysis, 37 species of subgenus *Leptostemonum* and two outgroup species (*Calystegia*, Convolvulaceae) were included, and their chloroplast genome sequences were obtained from the NCBI GenBank database. A total of 78 coding sequences (CDSs), representing single-copy genes shared across the sampled taxa, were selected for phylogenetic analysis. For genes duplicated in the inverted repeat (IR) regions, only one copy was retained. The selected CDSs were concatenated and aligned using MAFFT in the Geneious Prime program v.2025.2.2 (Katoh and Standley 2013). The analysis model was tested using PhyloSuite v.2.0.2 (Zhang et al. 2020; Xiang et al. 2023). The best model for maximum-likelihood (ML) analysis was constructed using ModelFinder v.3.0.1 (Kalyaanamoorthy et al. 2017). ML analysis was performed using IQ-tree v.2.2.0 (Nguyen et al. 2015) by setting the TVM+R3 + F model with 5,000 ultrafast bootstraps (Minh et al. 2013). The resulting phylogenetic tree was visualized using FigTree v.1.4.4 (http://tree.bio.ed.ac.uk/software/figtree). Finally, mVISTA (Frazer et al. 2004) was used to perform comparative chloroplast genome analysis using the chloroplast genome of *S. hieronymi* as a reference to identify variation hotspots among *S. carolinense*, its close relatives, and invasive *Solanum* species.

## Results

**Figure 2.**
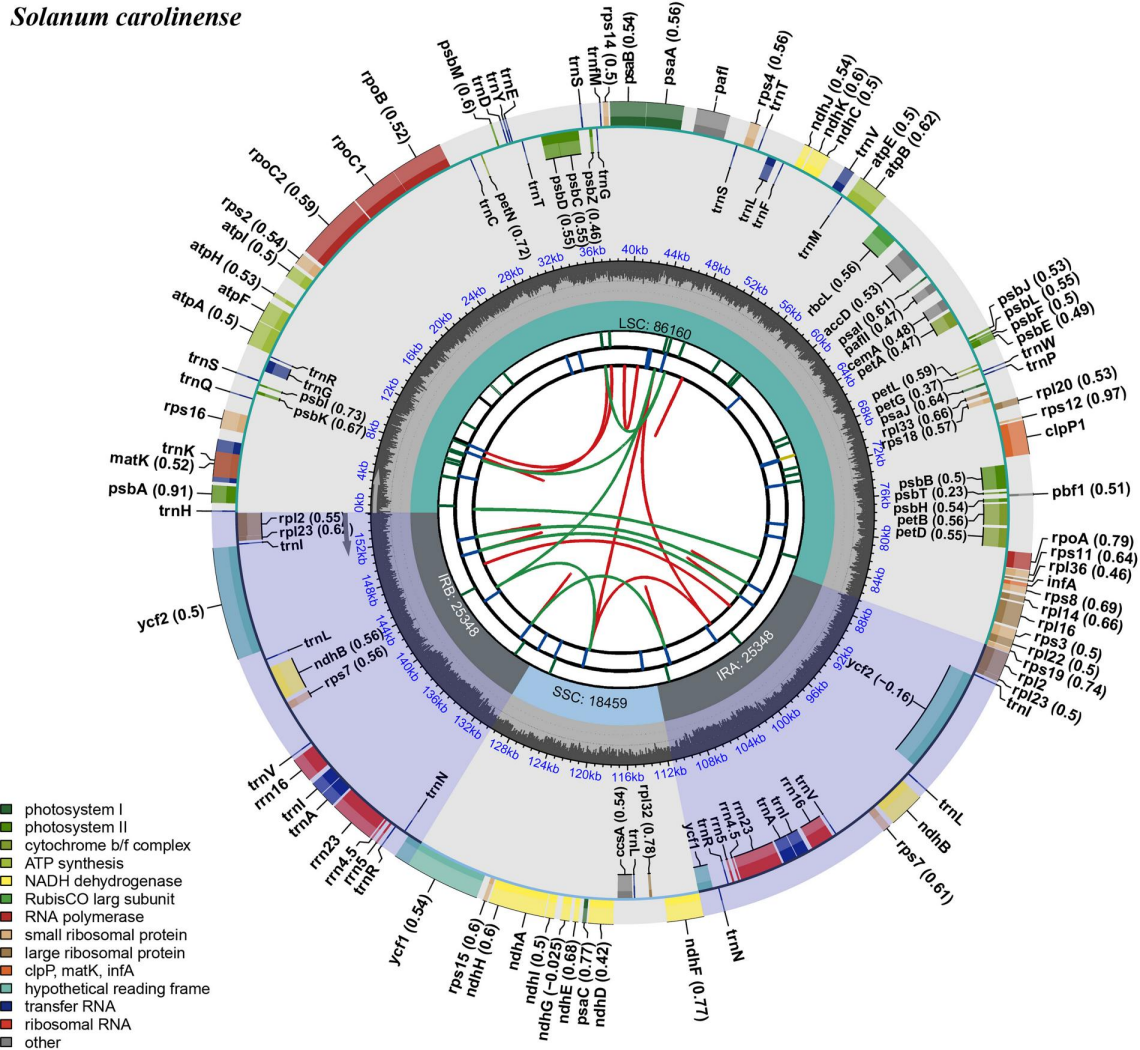
Circular map of the complete chloroplast genome of *solanum carolinense*. The map contains six distinct tracks arranged from the center outward. The innermost track shows dispersed repeats, connected by red and green arcs indicating forward and reverse directions, respectively. The second track highlights long tandem repeats as blue bands, followed by a third track marking short tandem repeats (microsatellites), represented as green bands. The fourth track shows the LSC, SSC, and IR regions. The fifth track depicts the GC content across the plastome. The outermost track displays genes as colored boxes, with inner boxes indicating clockwise transcription and outer boxes indicating counterclockwise transcription. Numbers in parentheses following gene names represent optional codon usage bias.

The complete chloroplast genome of *S. carolinense* has a total length of 155,315 bp and a 37.6% GC content (Figure 2), with an average read coverage depth of 306.66 X (Figure S2). The genome displayed a quadripartite structure comprising a pair of inverted repeats (IR, 25,348 bp each) separating a large single-copy region (LSC, 86,160 bp) from a small single-copy region (SSC, 18,459 bp). The complete genome sequence was deposited in GenBank under accession number PX547999.

The chloroplast genome comprised 129 genes, including 84 CDSs, 37 tRNA genes, 8 rRNA genes, and one pseudogene (*infA*). The IR contained 17 genes, including 6 CDS genes, 7 tRNA genes, and 4 rRNA genes (CDS: *ndhB*, *rpl2*, *rpl23*, *rps7*, *rps12*, *ycf2*; tRNA: *trnA-UGC*, *trnl-CAU*, *trnl-GAU*, *trnL-CAA*, *trnN-GUU*, *trnR-ACG*, *trnV-GAC*; rRNA: *rrn4.5*, *rrn5*, *rrn16*, *rrn23*). A total of 11 cis-splicing genes were observed; among these, nine genes contained a single intron (*atpF*, *ndhA*, *ndhB*, *petB*, *petD*, *rpl2*, *rpl16*, *rpoC1*, and *rps16*) while two genes contained two introns (*clpP1* and *pafI*) (Figure S3). The trans-spliced gene *rps12* had a single intron spanning the LSC and IR regions, with the 3′ end in the LSC region and the 5′ end in the IR region (Figure S4).

**Figure 3.**
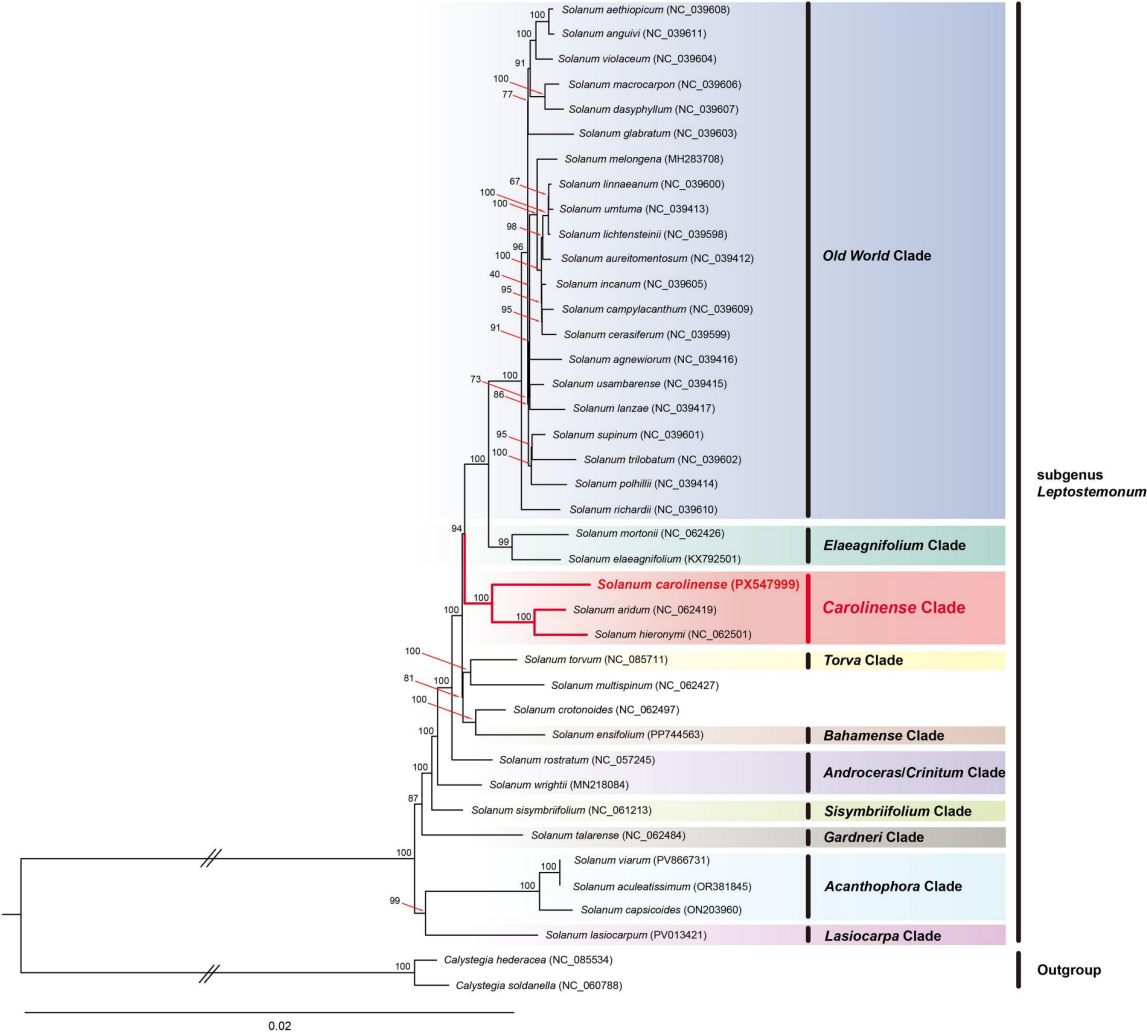
Maximum-likelihood phylogenetic tree of 38 species in the genus *solanum* (subgenus *leptostemonum*) with two outgroups (genus *calystegia*) based on concatenated 78 coding DNA sequences of chloroplast genomes. Numbers above the nodes indicate the bootstrap values: *Solanum aethiopicum* NC_039608 (Aubriot et al. 2018), *S. anguivi* NC_039611 (Aubriot et al. 2018), *S. violaceum* NC_039604 (Aubriot et al. 2018), *S. macrocarpon* NC_039606 (Aubriot et al. 2018), *S. dasyphyllum* NC_039607 (Aubriot et al. 2018), *S. glabratum* NC_039603 (Aubriot et al. 2018), *S. melongena* MH283708 (Aubriot et al. 2018), *S. linnaeanum* NC_039600 (Aubriot et al. 2018), *S. umtuma* NC_039413 (Aubriot et al. 2018), *S. lichtensteinii* NC_039598 (Aubriot et al. 2018), *S. aureitomentosum* NC_039412 (Aubriot et al. 2018), *S. incanum* NC_039605 (Aubriot et al. 2018), *S. campylacanthum* NC_039609 (Aubriot et al. 2018), *S. cerasiferum* NC_039599 (Aubriot et al. 2018), *S. agnewiorum* NC_039416 (Aubriot et al. 2018), *S. usambarense* NC_039415 (Aubriot et al. 2018), *S. lanzae* NC_039417 (Aubriot et al. 2018), *S. supinum* NC_039601 (Aubriot et al. 2018), *S. trilobatum* NC_039602 (Aubriot et al. 2018), *S. polhillii* NC_039414 (Aubriot et al. 2018), *S. richardii* NC_039610 (Aubriot et al. 2018), *S. mortonii* NC_062426 (Gagnon et al. 2022), *S. elaeagnifolium* KX792501 (Zhu et al. 2020), *S. carolinense* PX547999 (present study), *S. aridum* NC_062419 (Gagnon et al. 2022), *S. hieronymi* NC_062501 (Gagnon et al. 2022), *S. torvum* NC_085711 (Zhang et al. 2024), *S. multispinum* NC_062427 (Gagnon et al. 2022), *S. crotonoides* NC_062497 (Gagnon et al. 2022), *S. ensifolium* PP744563 (Graham et al. 2025), *S. rostratum* NC_057245 (Shi and Qiu 2020), *S. wrightii* MN218084 (Yang et al. 2023), *S. sisymbriifolium* NC_061213 (Yin et al. 2022), *S. talarense* NC_062484 (Gagnon et al. 2022), *S. viarum* PV866731 (Kim et al. 2025), *S. aculeatissimum* OR381845 (Zhang et al. 2024), *S. capsicoides* ON203960 (unpublished), *S. lasiocarpum* PV013421 (unpublished), *calystegia hederacea* NC_085534 (Fu et al. 2024), and *C. soldanella* NC_060788 (Wu et al. 2022).

ML phylogenetic analysis supported the monophyly of subgenus *Leptostemonum* (bootstrap value = 100) (Figure 3, Figure S5). Within the subgenus *Leptostemonum*, species were divided into ten clades. Furthermore, *S. carolinense* formed a strongly supported clade with *S. aridum* and *S. hieronymi* Kuntze (*Carolinense* clade) (bootstrap value = 100).

Comparative analysis of the chloroplast genomes revealed overall high sequence conservation among species, with coding regions more conserved than non-coding regions (Figure S6).

## Discussion and conclusion

In this study, we determined and analyzed the chloroplast genome sequence of *S. carolinense*. The chloroplast genome exhibited a typical quadripartite structure, as in most angiosperms (Kim et al. 2024), and pseudogenization of the chloroplast *infA* gene was also detected, a phenomenon known to occur repeatedly and independently across diverse angiosperm lineages (Millen et al. 2001).

The ML analysis results were consistent with those of previous phylogenetic studies of subgenus *Leptostemonum* (Levin et al. 2006; Stern et al. 2011; Zhang et al. 2013; Wahlert et al. 2014, 2015). *S. multispinum* N.E.Br. and *S. crotonoides* Lam. were exceptions because their phylogenetic relationships remained unresolved (Levin et al. 2006; Stern et al. 2011). *S. carolinense* was placed within the *Carolinense* clade (Levin et al. 2006; Zhang et al. 2013) and showed a close relationship with *S. aridum* (Stern et al. 2011; Wahlert et al. 2015) and *S. hieronymi* (Wahlert et al. 2014).

Comparative analysis of chloroplast genomes did not identify any structural variations directly associated with invasiveness. To clarify genetic features related to invasiveness, further verification will be required through comparative analyses including a broader range of invasive *Solanum* taxa or additional studies incorporating nuclear genome data.

Nonetheless, this study provides a comprehensive chloroplast genome dataset for *S. carolinense*, and these findings will serve as fundamental resources for studies on phylogeny, evolution, species identification, and invasion within *Solanum* and subgenus *Leptostemonum*.

## Supplementary Material

Supplemental Material

## Data Availability

The complete chloroplast genome sequence data for *S. carolinense* are available in GenBank of NCBI (https://www.ncbi.nlm.nih.gov/nuccore/PX547999) under accession number PX547999. The associated BioProject, Bio-Sample, and SRA numbers are PRJNA1348909, SAMN52921038, and SRR35877184, respectively.
