# Supplementary material for: The complete chloroplast genome sequence and phylogenetic analysis of an invasive plant Solanum carolinense Linnaeus (Solanaceae) in Korea
Source: Mitochondrial DNA B Resour. 2026 Feb 25;11(4):451–6. doi: 10.1080/23802359.2026.2635843 (PMC12943800; doi:10.1080/23802359.2026.2635843)
Supplement: Supplemental Material [file TMDN_A_2635843_SM7878.docx]

**
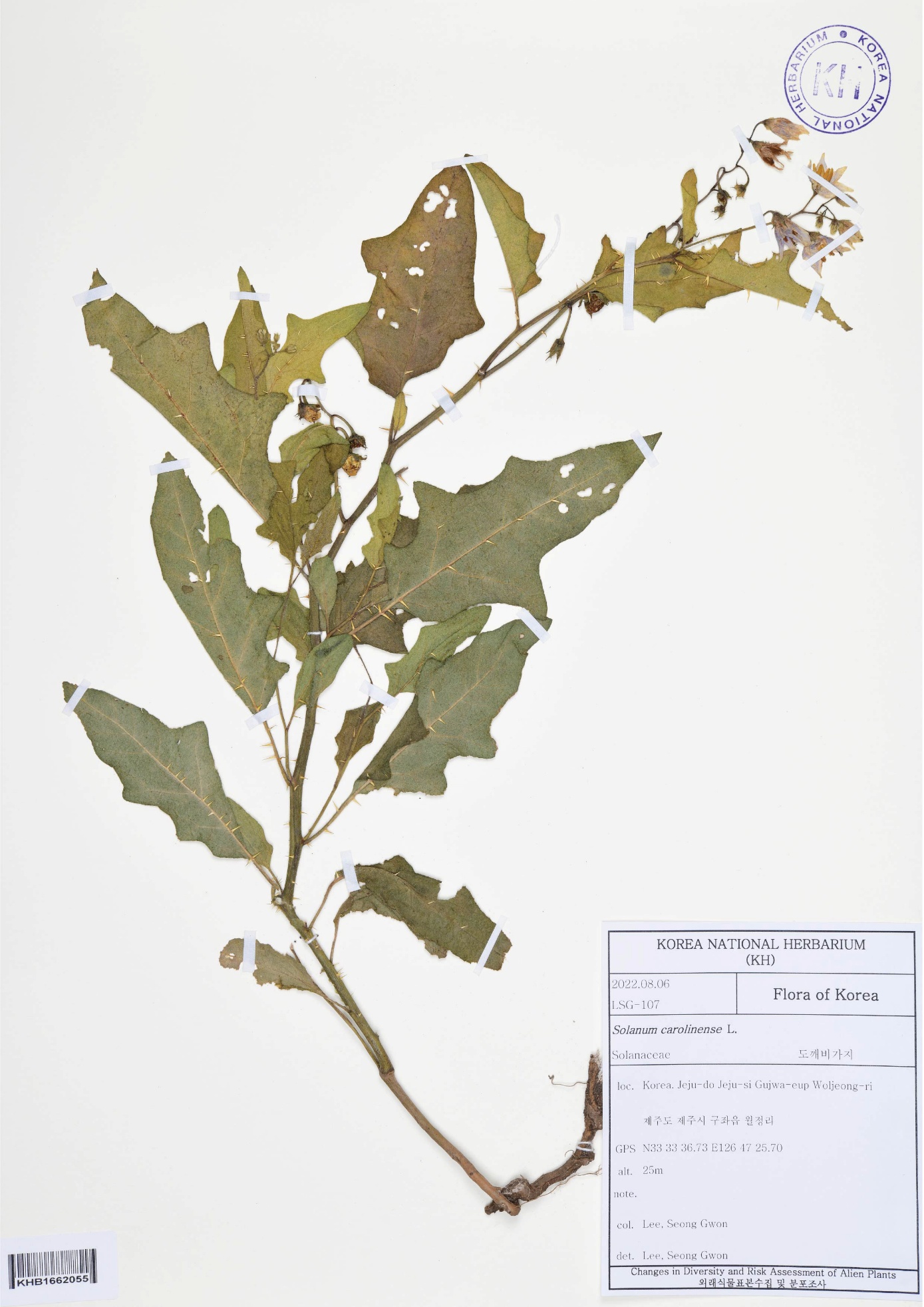
**

**Figure S1.** Korea National Arboretum herbarium collection specimens for *Solanum carolinense* Linnaeus (voucher number: KHB1662055).

**
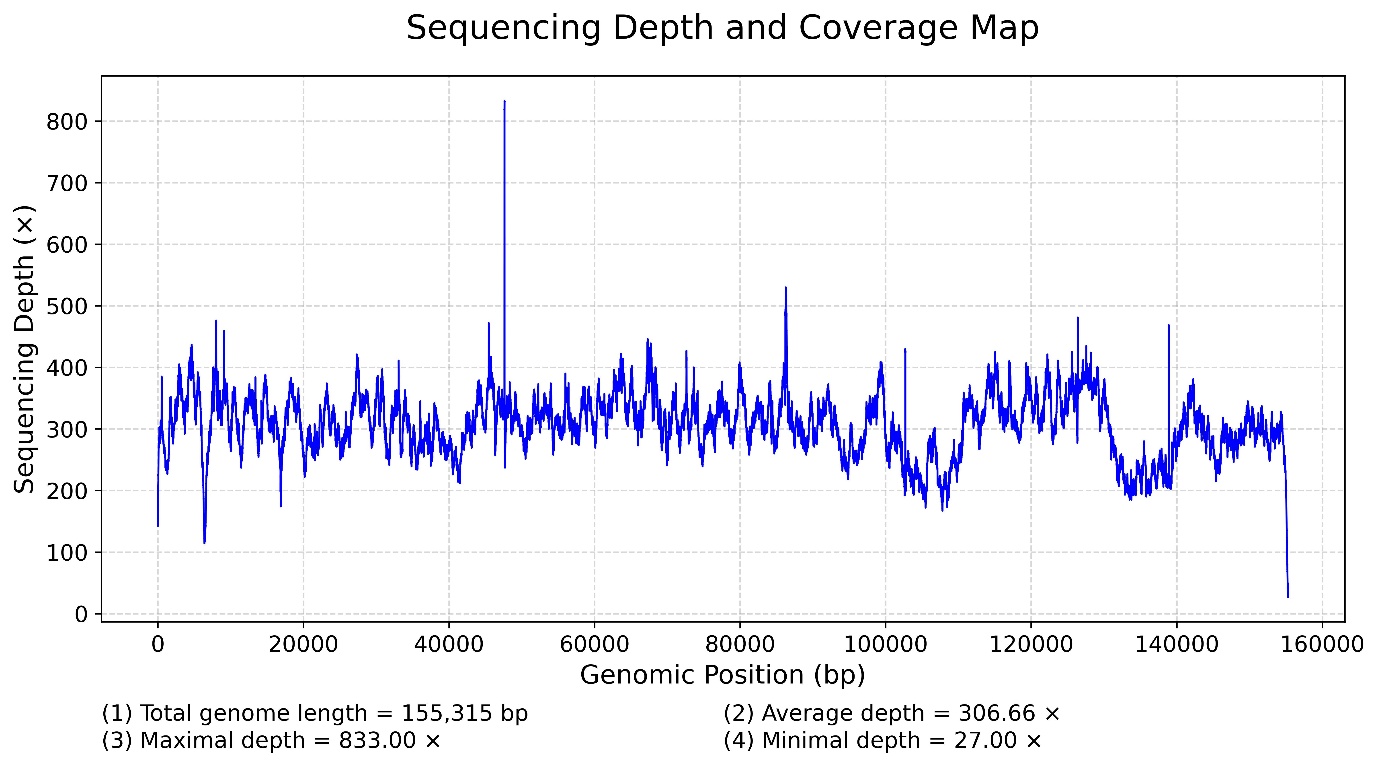
**

**Figure S2.** Sequencing depth and coverage map of the chloroplast genome assembly of *Solanum* *carolinense*. The horizontal axis represents the base position of the plastome, and the vertical axis indicates the sequencing depth corresponding to each base. This figure was generated using the Draw_SequencingDepth.py script provided by Ni et al. (2023).


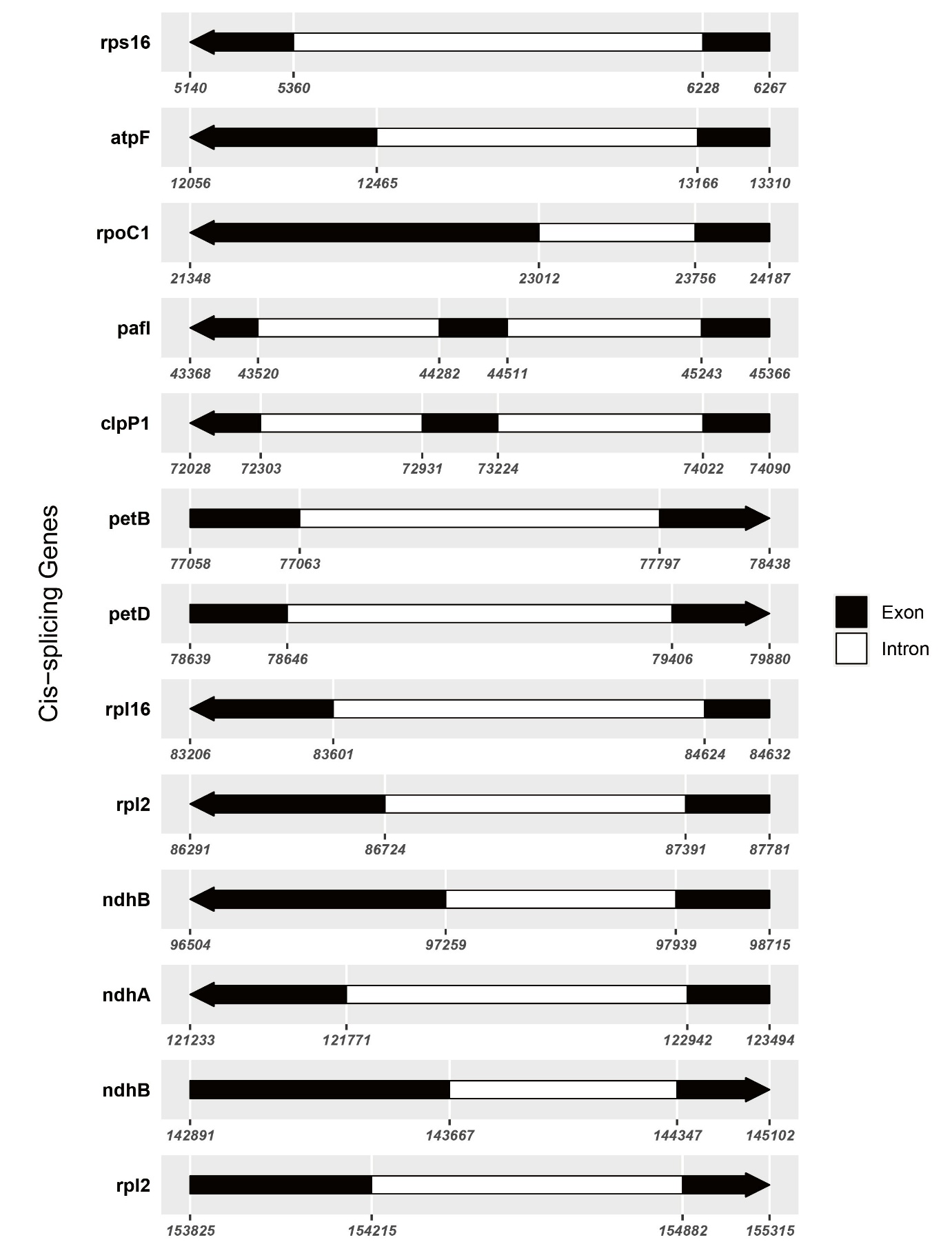


**Figure S3.** Schematic of cis-spliced genes in the chloroplast genome of *Solanum carolinense*.

**
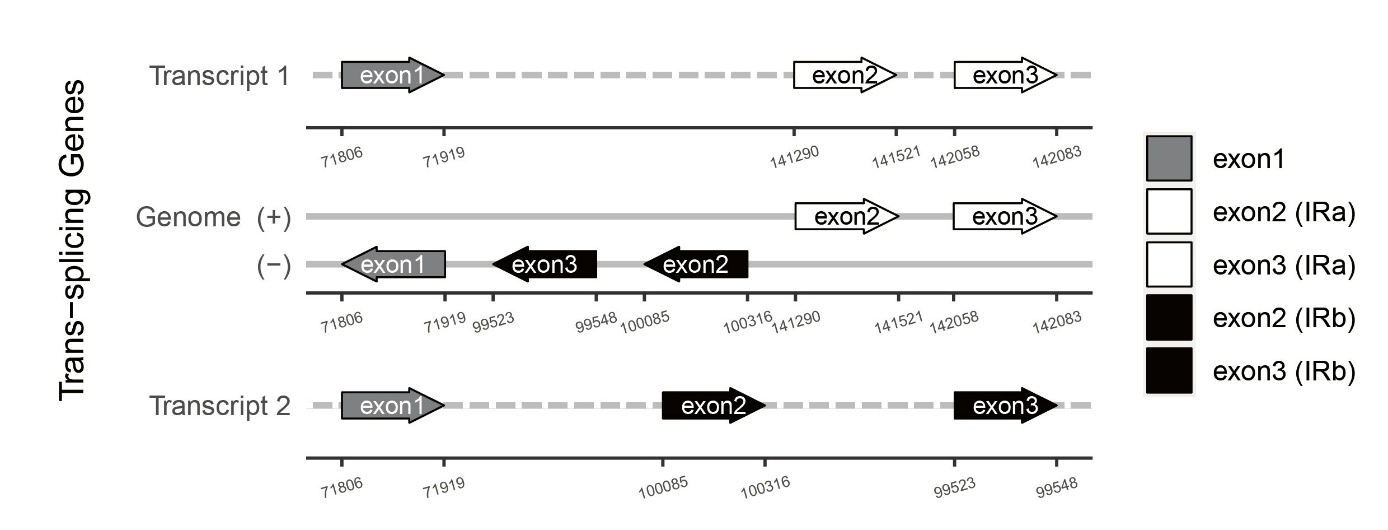
**

**Figure S4.** Schematic of the trans-spliced gene *rps12* in the chloroplast genome of *Solanum carolinense*.

**
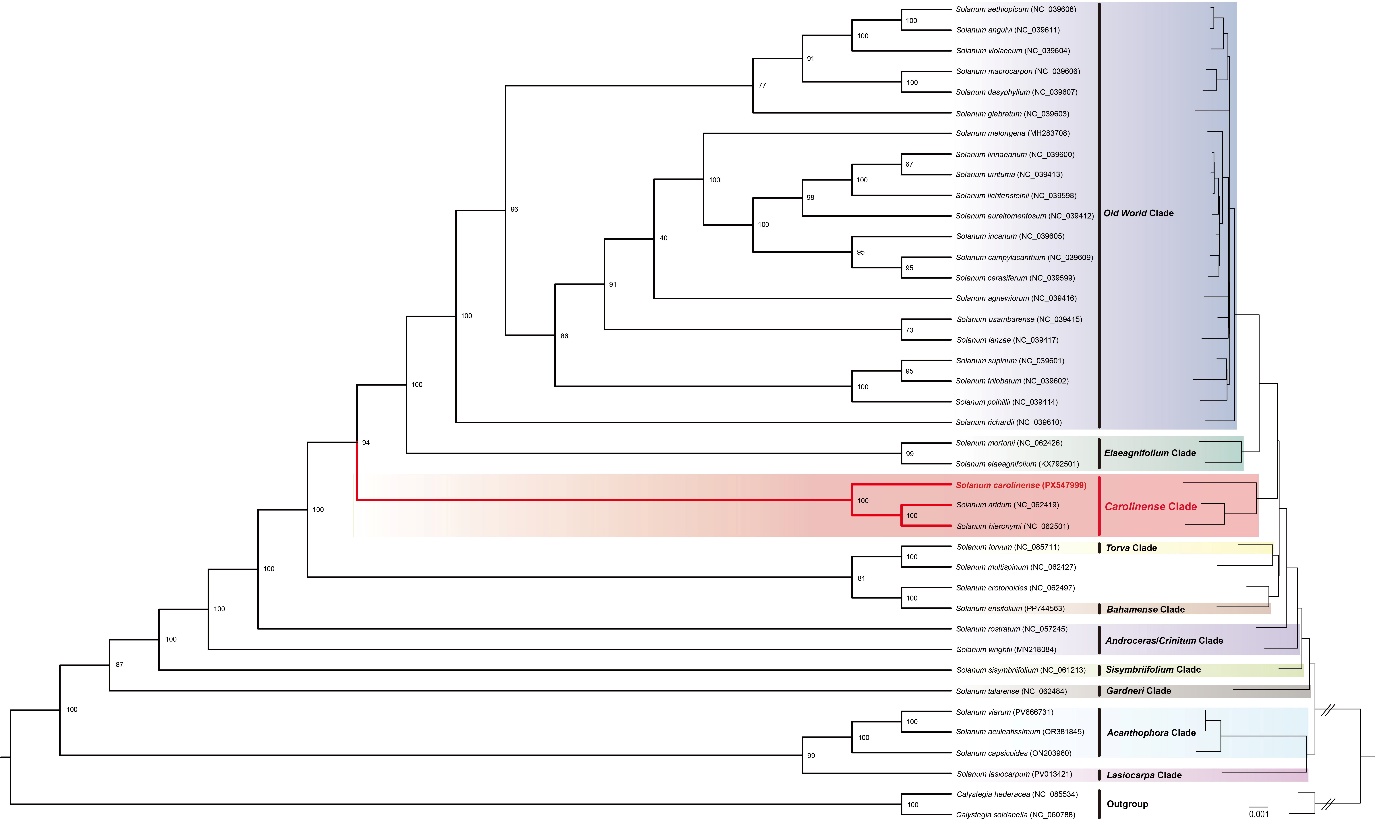
**

**Figure S5.** Phylogenetic tree (left in cladogram style, right with branch lengths) inferred from chloroplast genome data. Maximum likelihood phylogeny illustrating the phylogenetic relationships of 38 species in the genus *Solanum* (subgenus *Leptostemonum*) with two outgroups (genus *Calystegia*) based on concatenated 78 coding DNA sequences of chloroplast genomes. Numbers above the nodes indicate the bootstrap values: *Solanum aethiopicum* NC_039608 (Aubriot et al. 2018), *S*. *anguivi* NC_039611 (Aubriot et al. 2018), *S*. *violaceum* NC_039604 (Aubriot et al. 2018), *S*. *macrocarpon* NC_039606 (Aubriot et al. 2018), *S*. *dasyphyllum* NC_039607 (Aubriot et al. 2018), *S*. *glabratum* NC_039603 (Aubriot et al. 2018), *S*. *melongena* MH283708 (Aubriot et al. 2018), *S*. *linnaeanum* NC_039600 (Aubriot et al. 2018), *S*. *umtuma* NC_039413 (Aubriot et al. 2018), *S*. *lichtensteinii* NC_039598 (Aubriot et al. 2018), *S*. *aureitomentosum* NC_039412 (Aubriot et al. 2018), *S*. *incanum* NC_039605 (Aubriot et al. 2018), *S*. *campylacanthum* NC_039609 (Aubriot et al. 2018), *S*. *cerasiferum* NC_039599 (Aubriot et al. 2018), *S*. *agnewiorum* NC_039416 (Aubriot et al. 2018), *S*. *usambarense* NC_039415 (Aubriot et al. 2018), *S*. *lanzae* NC_039417 (Aubriot et al. 2018), *S*. *supinum* NC_039601 (Aubriot et al. 2018), *S*. *trilobatum* NC_039602 (Aubriot et al. 2018), *S*. *polhillii* NC_039414 (Aubriot et al. 2018), *S*. *richardii* NC_039610 (Aubriot et al. 2018), *S*. *mortonii* NC_062426 (Gagnon et al. 2022), *S*. *elaeagnifolium* KX792501 (Zhu et al. 2020), *S*. *carolinense* PX547999 (present study), *S*. *aridum* NC_062419 (Gagnon et al. 2022), *S*. *hieronymi* NC_062501 (Gagnon et al. 2022), *S*. *torvum* NC_085711 (Zhang et al. 2024), *S*. *multispinum* NC_062427 (Gagnon et al. 2022), *S*. *crotonoides* NC_062497 (Gagnon et al. 2022), *S*. *ensifolium* PP744563 (Graham et al. 2025), *S*. *rostratum* NC_057245 (Shi and Qiu 2020), *S*. *wrightii* MN218084 (Yang et al. 2023), *S*. *sisymbriifolium* NC_061213 (Yin et al. 2022), *S*. *talarense* NC_062484 (Gagnon et al. 2022), *S*. *viarum* PV866731 (Kim et al. 2025), *S*. *aculeatissimum* OR381845 (Zhang et al. 2024), *S*. *capsicoides* ON203960 (unpublished), *S*. *lasiocarpum* PV013421 (unpublished), *Calystegia* *hederacea* NC_085534 (Fu et al. 2024), and *C*. *soldanella* NC_060788 (Wu et al. 2022).

**
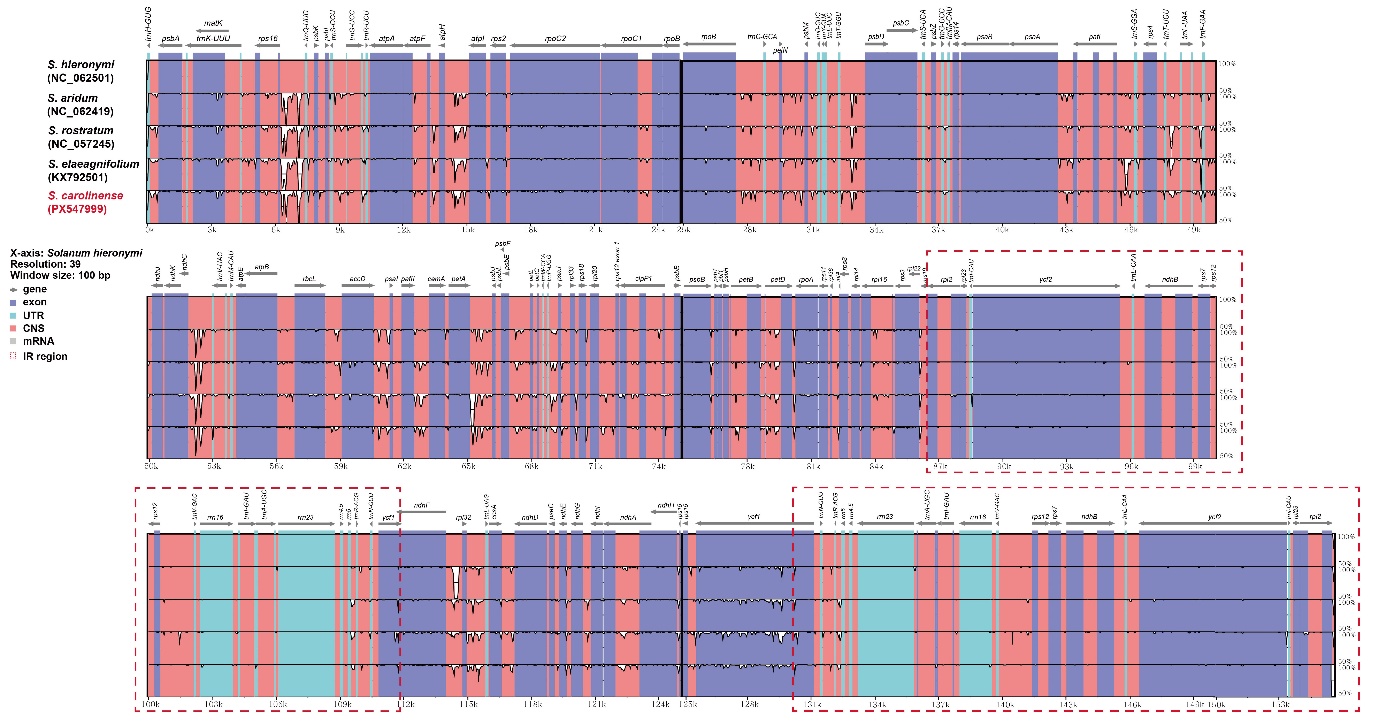
**

**Figure S6.** Aligned sequence plots were generated for the chloroplast genomes of five species in subgenus *Leptostemonum*, including close relatives (*S.* *hieronymi* and *S*. *aridum*), invasive *Solanum* species (*S*. *rostratum* and *S*. *elaeagnifolium*), and the focal species *S*. *carolinense*, using the chloroplast genome of *S*. *hieronymi* as the reference: *Solanum hieronymi* NC_062501 (Gagnon et al. 2022), *S*. *aridum* NC_062419 (Gagnon et al. 2022), *S*. *rostratum* NC_057245 (Shi and Qiu 2020), *S*. *elaeagnifolium* KX792501 (Zhu et al. 2020), *S*. *carolinense* PX547999 (present study).

**Supplemental material**

**Figure S1.** Korea National Arboretum herbarium collection specimens for *Solanum carolinense* Linnaeus (voucher number: KHB1662055).

**Figure S2.** Sequencing depth and coverage map of the chloroplast genome assembly of *Solanum* *carolinense*. The horizontal axis represents the base position of the plastome, and the vertical axis indicates the sequencing depth corresponding to each base. This figure was generated using the Draw_SequencingDepth.py script provided by Ni et al. (2023).

**Figure S3.** Schematic of cis-spliced genes in the chloroplast genome of *Solanum carolinense*.

**Figure S4.** Schematic of the trans-spliced gene *rps12* in the chloroplast genome of *Solanum carolinense*.

**Figure S5.** Phylogenetic tree (left in cladogram style, right with branch lengths) inferred from chloroplast genome data. Maximum likelihood phylogeny illustrating the phylogenetic relationships of 38 species in the genus *Solanum* (subgenus *Leptostemonum*) with two outgroups (genus *Calystegia*) based on concatenated 78 coding DNA sequences of chloroplast genomes. Numbers above the nodes indicate the bootstrap values: *Solanum aethiopicum* NC_039608 (Aubriot et al. 2018), *S*. *anguivi* NC_039611 (Aubriot et al. 2018), *S*. *violaceum* NC_039604 (Aubriot et al. 2018), *S*. *macrocarpon* NC_039606 (Aubriot et al. 2018), *S*. *dasyphyllum* NC_039607 (Aubriot et al. 2018), *S*. *glabratum* NC_039603 (Aubriot et al. 2018), *S*. *melongena* MH283708 (Aubriot et al. 2018), *S*. *linnaeanum* NC_039600 (Aubriot et al. 2018), *S*. *umtuma* NC_039413 (Aubriot et al. 2018), *S*. *lichtensteinii* NC_039598 (Aubriot et al. 2018), *S*. *aureitomentosum* NC_039412 (Aubriot et al. 2018), *S*. *incanum* NC_039605 (Aubriot et al. 2018), *S*. *campylacanthum* NC_039609 (Aubriot et al. 2018), *S*. *cerasiferum* NC_039599 (Aubriot et al. 2018), *S*. *agnewiorum* NC_039416 (Aubriot et al. 2018), *S*. *usambarense* NC_039415 (Aubriot et al. 2018), *S*. *lanzae* NC_039417 (Aubriot et al. 2018), *S*. *supinum* NC_039601 (Aubriot et al. 2018), *S*. *trilobatum* NC_039602 (Aubriot et al. 2018), *S*. *polhillii* NC_039414 (Aubriot et al. 2018), *S*. *richardii* NC_039610 (Aubriot et al. 2018), *S*. *mortonii* NC_062426 (Gagnon et al. 2022), *S*. *elaeagnifolium* KX792501 (Zhu et al. 2020), *S*. *carolinense* PX547999 (present study), *S*. *aridum* NC_062419 (Gagnon et al. 2022), *S*. *hieronymi* NC_062501 (Gagnon et al. 2022), *S*. *torvum* NC_085711 (Zhang et al. 2024), *S*. *multispinum* NC_062427 (Gagnon et al. 2022), *S*. *crotonoides* NC_062497 (Gagnon et al. 2022), *S*. *ensifolium* PP744563 (Graham et al. 2025), *S*. *rostratum* NC_057245 (Shi and Qiu 2020), *S*. *wrightii* MN218084 (Yang et al. 2023), *S*. *sisymbriifolium* NC_061213 (Yin et al. 2022), *S*. *talarense* NC_062484 (Gagnon et al. 2022), *S*. *viarum* PV866731 (Kim et al. 2025), *S*. *aculeatissimum* OR381845 (Zhang et al. 2024), *S*. *capsicoides* ON203960 (unpublished), *S*. *lasiocarpum* PV013421 (unpublished), *Calystegia* *hederacea* NC_085534 (Fu et al. 2024), and *C*. *soldanella* NC_060788 (Wu et al. 2022).

**Figure S6.** Aligned sequence plots were generated for the chloroplast genomes of five species in subgenus *Leptostemonum*, including close relatives (*S.* *hieronymi* and *S*. *aridum*), invasive *Solanum* species (*S*. *rostratum* and *S*. *elaeagnifolium*), and the focal species *S*. *carolinense*, using the chloroplast genome of *S*. *hieronymi* as the reference: *Solanum hieronymi* NC_062501 (Gagnon et al. 2022), *S*. *aridum* NC_062419 (Gagnon et al. 2022), *S*. *rostratum* NC_057245 (Shi and Qiu 2020), *S*. *elaeagnifolium* KX792501 (Zhu et al. 2020), *S*. *carolinense* PX547999 (present study).
